# Supplementary material for: Total biosynthesis of the cyclic AMP booster forskolin from Coleus forskohlii
Source: eLife. 2017 Mar 14;6:e23001. doi: 10.7554/eLife.23001 (PMC5388535; doi:10.7554/eLife.23001)
Supplement: Figure 1—source data 1. — DOI: http://dx.doi.org/10.7554/eLife.23001.004 [file elife-23001-fig1-data1.docx]

**Figure 1-source data 1.**

cDNAs identified in the *C. forskohlii* root cork transcriptome and cloned during this work, with the GeneBank accession numbers

| **Gene Name** | **Accession Number** |
| --- | --- |
| ***Cf*CYP716A67** | KT382331 |
| ***Cf*CYP716A68** | KT382332 |
| ***Cf*CYP716A69** | KT382333 |
| ***Cf*CYP716C10** | KT382334 |
| ***Cf*CYP716D20** | KT382335 |
| ***Cf*CYP707A90** | KT382350 |
| ***Cf*CYP71D377** | KT382339 |
| ***Cf*CYP71D379** | KT382338 |
| ***Cf*CYP71D380** | KT382337 |
| ***Cf*CYP71D381** | KT382342 |
| ***Cf*CYP71D382** | KT382340 |
| ***Cf*CYP71D383** | KT382341 |
| ***Cf*CYP82D63** | KT382351 |
| ***Cf*CYP71BE32** | KT382343 |
| ***Cf*CYP89A110** | KT382357 |
| ***Cf*CYP96A70** | KT382356 |
| ***Cf*CYP72A293** | KT382344 |
| ***Cf*CYP72A294** | KT382345 |
| ***Cf*CYP71AU43** | KT382355 |
| ***Cf*CYP71AT85** | KT382353 |
| ***Cf*CYP71AT86** | KT382352 |
| ***Cf*CYP71AT87** | KT382354 |
| ***Cf*CYP76AH8** | KT382348 |
| ***Cf*CYP76AH9** | KT382347 |
| ***Cf*CYP76AH10** | KT382346 |
| ***Cf*CYP76AH11** | KT382349 |
| ***Cf*CYP76AH15** | KT382358 |
| ***Cf*CYP76AH16** | KT382359 |
| ***Cf*CYP76AH17** | KT382360 |
| ***Cf*CPR** | KX151181 |
| ***Cf*ACT1-6** | KT382361 |
| ***Cf*ACT1-8** | KT382363 |
| ***Cf*ACT2** | KT382364 |
| ***Cf*ACT3** | KT382365 |
| ***Cf*ACT4** | KT382366 |
| ***Cf*ACT5** | KT382367 |
| ***Cf*ACT6** | KT382368 |
| ***Cf*ACT7** | KT382369 |
| ***Cf*ACT8** | KT382370 |
