# Supplementary material for: Total biosynthesis of the cyclic AMP booster forskolin from Coleus forskohlii
Source: eLife. 2017 Mar 14;6:e23001. doi: 10.7554/eLife.23001 (PMC5388535; doi:10.7554/eLife.23001)
Supplement: Figure 1—source data 2. — cDNAs involved in terpenoid metabolism are marked in bold. DOI: http://dx.doi.org/10.7554/eLife.23001.005 [file elife-23001-fig1-data2.docx]

**Figure 1-source data 2.**

Table of FPKM (Fragments Per Kilobase of transcript per Million mapped reads) values of the first 20 most abundant cDNAs identified in the root cork transcriptome library. cDNAs involved in terpenoid metabolism are marked in bold.

|  | **Gene description** | **Gene** | **FPKM** | **Contigs** |
| --- | --- | --- | --- | --- |
| 1 | ribosomal RNA | comp25558 | 39920,88 | comp23553_c0_seq1,comp23553_c0_seq2 |
| 2 | heat shock protein | comp25441_c0 | 18238,91 | comp25441_c0_seq1,comp25441_c0_seq2 |
| 3 | ribosomal RNA | comp25645_c0 | 15709,42 | comp25645_c0_seq1,comp25645_c0_seq2 |
| 4 | metallothionein-like protein (MT1) | comp25436_c0 | 13658,41 | comp25436_c0_seq1 |
| 5 | methionine adenosyltransferase (MAT) | comp25906_c0 | 12209,92 | comp25906_c0_seq1,comp25906_c0_seq2 |
| 6 | pleiotropic drug resistance protein 2 | comp27718_c0 | 11833,07 | comp27718_c0_seq1,comp27718_c0_seq2 |
| 7 | ribosomal RNA | comp27982_c0 | 11709,69 | comp27982_c0_seq1,comp27982_c0_seq10 |
| 8 | dehydration-responsive protein RD22 | comp10913_c0 | 9385,41 | comp10913_c0_seq1,comp10913_c0_seq2 |
| **9** | **P450 (CYP76AH15, CYP76AH8)** | **comp25558_c0** | **7690,25** | comp25558_c0_seq1,comp25558_c0_seq2 |
| 10 | lipoyl synthase 1 | comp10915_c0 | 7554,82 | comp10915_c0_seq1 |
| 11 | zinc finger CCCH domain-containing protein | comp23355_c0 | 6772,93 | comp23355_c0_seq1,comp23355_c0_seq2 |
| 12 | Chaperone protein dnaJ | comp26059_c0 | 6057 | comp26059_c0_seq1,comp26059_c0_seq2 |
| 13 | elongation factor 1-alpha | comp27357_c0 | 5912,26 | comp27357_c0_seq1,comp27357_c0_seq2 |
| 14 | calcium ATPase | comp27655_c1 | 5911,83 | comp27655_c1_seq1,comp27655_c1_seq10 |
| **15** | **DXS2** | **comp26013_c0** | **5901,04** | comp26013_c0_seq1,comp26013_c0_seq2 |
| **16** | **beta farnesene synthase** | **comp26811_c0** | **5661,04** | comp26811_c0_seq1 |
| 17 | helicase like protein | comp27987_c0 | 5568,13 | comp27987_c0_seq1,comp27987_c0_seq10 |
| 18 | S-adenosylmethionine decarboxylase | comp28104_c0 | 5231 | comp28104_c0_seq1 |
| 19 | transcription factor ILR3 | comp25820_c4 | 5103,94 | comp25820_c4_seq1,comp25820_c4_seq10 |
| 20 | galactinol--sucrose galactosyltransferase (galactose metabolism) | comp16708_c0 | 5041,74 | comp16708_c0_seq1,comp16708_c0_seq2 |
