# Supplementary material for: Total biosynthesis of the cyclic AMP booster forskolin from Coleus forskohlii
Source: eLife. 2017 Mar 14;6:e23001. doi: 10.7554/eLife.23001 (PMC5388535; doi:10.7554/eLife.23001)
Supplement: Figure 1—source data 3. — Construction of plasmids for expression of CfTPS2, CfTPS3, CfTPS1 is described in Andersen-Ranberg et al. (2016). U (uracil, marked in bold), represents the cleavage site, used in the USER cloning (Nour-Eldin et al., 2006). DOI: http://dx.doi.org/10.7554/eLife.23001.006 [file elife-23001-fig1-data3.docx]

**Figure 1-source data 3.**

Table of primers used in this study. Construction of plasmids for expression of *Cf*TPS2, *Cf*TPS3, *Cf*TPS1 is described in (***Andersen-Ranberg et al.,* *2016***). U (uracil, marked in bold), represents the cleavage site, used in the USER cloning (***Nour-Eldin et al.,* *2006***)

| **For FL cDNAs' cloning** | | | |
| --- | --- | --- | --- |
| **Gene Name** | | **primer name** | **sequence** |
| CfCYP707A90 | | CYP707A90_For | ATGGAACTCATCTGTTTGTTGTTGATTG |
|  | | CYP707A90_Rev | TCATTTCTTGAGAGAGATTTTGATGG |
| CfCYP96A70 | | CYP96A70_For | ATGGAGTCATTTTCATTGCTGTATCC |
|  | | CYP96A70_Rev | TCAAGCCCACCTCTTGCTAACCC |
| CfCYP76AH11 | | CYP76AH11_For | ATGGAGTTGGTGCAAGTGATTGCGG |
|  | | CYP76AH11_Rev | TCAAATAGAAGGTGGGTTGAGTGGG |
| CfCYP89A110 | | CYP89A110_For | ATGATGAATTATCCACCCATTTTCG |
|  | | CYP89A110_Rev | TTAACTTGTTCTGGGAGAGATGCGG |
| CYP76AH10 | | CYP76AH10.For | ATGGATAACTTTGTGCTCCTCG |
|  | | CYP76AH10.Rev | TCAGAGGCTGATGGGGGTGGCTC |
| CYP76AH9 | | CYP76AH9.For | ATGGATTTCTTCACTCTTCTCGCTGC |
|  | | CYP76AH9.Rev | TCAAGGTTGGATGGGATAGGCCCTG |
| CYP76AH8 | | CYP76AH8.For | ATGGAAACCATCACTCTTCTCC |
|  | | CYP76AH8.Rev | TCATGGCTTAAGTGGAATGATCC |
| CfCYP71AT86 | | CYP71AT86.For | ATGATAATTTATTTCTTGATAATTCTGCCAC |
|  | | CYP71AT86.Rev | CCAAACCTAACCAACATATTTCTTAGC |
| CfCYP71AT85 | | CYP71AT85.For | ATGATTTTTCTCTTCATGTCATTATTTCTTCC |
|  | | CYP71AT85.Rev | CTAAAATATATATTTCGTAGGGACAAGC |
| CfCYP71D382 | | CYP71D382.For | ATGGAGTCTCTCACTCTTGCTCTCTC |
|  | | CYP71D382.Rev | TCACTCAACGACCTTGAGAGGCC |
| CfCYP71AU43 | | CYP71AU43.For | ATGCTTCTGTTGAATCCGTTTCTGC |
|  | | CYP71AU43.Rev | TCAGATGCGAGTTGCAACAGCGAG |
| CfCYP71AT87 | | CYP71AT87.For2 | CAACTTAAAGATGATGCAGTTGGTCGCAGC |
|  | | CYP71AT87.Rev2 | TCACATAATGGCTGAGGATGGTGGAAGG |
| CfCYP728B26 | | CYP728B26.For | ATGATCATGATCTCCCTCCTCTGC |
|  | | CYP728B26.Rev | TTATAACAGTTTGTCTCTATGTTGCAAC |
| CfCYP716A67 | | CYP716A67.For | ATGGAGGCTTTCTACGTTTCTCTTTG |
|  | | CYP716A67.Rev | CTAAGGATTGTGAGGGAAGAGTCGAATG |
| CfCYP716A68 | | CYP716A68.For | CCTCCACCTGTGTATGGGATCACAC |
|  | | CYP716A68.Rev | TTAAGCTTGGTGGGGGTAGAGC |
| CfCYP716A69 | | CYP716A69.For | ATGGAGCTCTTCTATGCCTCTGTC |
|  | | CYP716A69.Rev | TCAAGGGTGGTTGTGGGGG |
| CfCYP716C10 | | CYP716C10.For | TCCAACCATGGAAATCTTGACGCTT |
|  | | CYP716C10.Rev | AGATTAATGGTGGTAGAGGCGGACT |
| CfCYP716D20 | | CYP716D20.For | ATGGAGGCCATTTGGATCATCACT |
|  | | CYP716D20.Rev | TTATATTAACGAGAGTGAATACGAATGGG |
| CfCYP71D379 | | CYP71D379.For | ATGGATTTCTCATTTTTCTGGAACTTCAC |
|  | | CYP71D379.Rev | TTAAGGCGACTGCTTGTAAGGGG |
| CfCYP71D383 | | CYP71D383.For | ATGGAGTTTGAGTTGGCATCATCATC |
|  | | CYP71D383.Rev | ATCAGGCGTGCAAAGGGCGC |
| CfCYP71D381 | | CYP71D381.For | ATGGAGTTCGATTTCCCGTCAGC |
|  | | CYP71D381.Rev | TCATGGAGCAAGTCGCAAAGGCC |
| CfCYP71BE32 | | CYP71BE32.For | CATCGTCATGGAGATCCAGTTTCC |
|  | | CYP71BE32.Rev | GCTGCAGTATCAGACAGGCAAAGG |
| CfCYP71D377 | | CYP71D377.For | ATGGAGCTGCCCTTGAGCTTCACCACTCTC |
|  | | CYP71D377.Rev | TTAAGATGAGATAGGAGCGGTTGGGACC |
| CfCYP72A293 | | CYP72A293.For | ATGGAGATATTATACAGTTGGGTGG |
|  | | CYP72A293.Rev | CTAATGGAGTGCGTGGAGGATCAAGG |
| CfCYP72A294 | | CYP72A294.For | ATGGAAATGGAAGTATTGTTATGCC |
|  | | CYP72A294.Rev | TTACAATTTGTGTAAAATCAAGTGAGCACC |
| CfCYP82D63 | | CYP82D63.For | ATGGAATTCCACTACACAATCTATGG |
|  | | CYP82D63.Rev | TTAGCTAATCGAATATAGAGTAGGAGCC |
| CfCYP76AH15 | | CYP76AH15.For | ATGGAAACCATGACTCTTCTCCTCC |
|  | | CYP76AH15.Rev | TCATGGCTTAAGTGGAATGATCCTG |
| CfCYP76AH16 | | CYP76AH16.For | ATGGAGTTGGTGGAAGTGATTGTGG |
|  | | CYP76AH16.rev | TCAAAATGAAGGGTTGAGTGGGATGAGG |
| CfCYP76AH17 | | CYP76AH17.For | ATGGAAAGCATGAATGCTCTTGTCGTCGG |
|  | | CYP76AH17.Rev | TTAAGGCTGAAGTGGAATGATCCTGAGG |
| CfCYP71D380 | | CYP71D380.For | ATGGATTTCTCATTTTTCTGGAACTTCAC |
|  | | CYP71D380.Rev | TTAAGGCGACTGCTTGTAAGGGG |
| CfCYP716E1 | | CfCYP716E1.For | GCTCGTTTTCTTAACCTTCAACTTCATGCC |
|  | | CfCYP716E1.Rev | GTGGCCTATTTTGGATGTGGGCG |
| CfCPR | | CPR.For | ATGGAATCGACTATTGAGAAGCTTTC |
|  | | CPR.rev | TCACCACACGTCACGCAGGTACCGG |
| RoCYP76AH4 | | CYP76AH4.For | ATGGATTCCTTTCCTCTTCTCGTTGC |
|  | | CYP76AH4.Rev | TCAAGACTTAACTATTGGGATAATCCTAAGC |
| CfACT1-6 | | ACT1.For | ATGAAGGTGGAAAGATTTAGCAGG |
|  | | ACT1.Rev | TTAGGTAGTAAATAATTTGATTTCTTCATCTTGC |
| CfACT1-7 | | ACT1.For | ATGAAGGTGGAAAGATTTAGCAGG |
|  | | ACT1.Rev | TTAGGTAGTAAATAATTTGATTTCTTCATCTTGC |
| CfACT1-8 | | ACT1-8.For | ATGAAGGTTGAAAGAATAAGCAGG |
|  | | ACT1-8.Rev | TTAGGTAGTAATTAATAATTTGATTTCTTCATC |
| CfACT2 | | ACT2.For | ATGATCCATTTACACAAAACAGTGTATCTCC |
|  | | ACT2.Rev | TTAGGTAGTAAATAATTTGATTTCTTCATCTTG |
| CfACT3 | | ACT3.For | ATGGAAGGCAAGGGTGTGGTTGTG |
|  | | ACT3.Rev | TCAAGAGTTGGAAGTGGGGATGAGTTG |
| CfACT4 | | ACT4.For | ATGAAGGTGGAGATAGAGAGCCAAAAGC |
|  | | ACT4.Rev | ATCTTAGATGTATGTAGCTGCTGAAAGTTG |
| CfACT5 | | ACT5.For | ATGGAGATCGAAACCCAAGTTATCTCTAC |
|  | | ACT5.Rev | TTATGAAATCAAAGAAGTTTTGTGATGTG |
| CfACT6 | | ACT6.For | ATGGTCGGAAAAATGAAGGTGGAGGTG |
|  | | ACT6.Rev | TCAGCCGGTGAGGATGCGAAGCTC |
| CfACT7 | | ACT7.For | ATGGCGACAGTCGAAACGATATCC |
|  | | ACT7.Rev | TTAATTATGAAGTTCAAAAATGTCATCACTGTC |
| CfACT8 | | ACT8.For | ATGGAAACATTAAACGTCAAGGTTTCG |
|  | | ACT8.Rev | TCACAGCGCCGATGTGATGAACAC |
| **For qPCRs** | |  |  |
| **Gene Name** | | **primer name** | **sequence** |
| CfCYP76AH8 | | CYP76AH8.qPCR.FOR1 | GCTGAAGAGCGTGGTAGGAGACG |
|  | | CYP76AH8.qPCR.REV1 | AGCCCAAAGAGCTCTCCTTTGTG |
| CfCYP76AH9 | | CYP76AH9.qPCR.FOR1 | ATCCTTCGCGAATCGAATGCTGCCC |
|  | | CYP76AH9.qPCR.REV1 | ATATGATAAGATCAAGGTTGGATG |
| CfCYP76AH10 | | CYP76AH10.qPCR.FOR1 | AATTCGAGGTGGGAGCTGAGGGG |
|  | | CYP76AH10.qPCR.REV1 | CACATAATGAATGACAACATACC |
| CfCYP76AH11 | | CYP76AH11.qPCR.FOR1 | CAATGTAACAACGAGTTCTTCACC |
|  | | CYP76AH11.qPCR.REV1 | GAAGAAGATCCTGAGGATGATCG |
| CfCYP76AH15 | | CYP76AH15.qPCR.FOR1 | TAGGAGAAAAGAAAGTGGTGAG |
|  | | CYP76AH15.qPCR.REV1 | GATGGCCAAGCCGAAGGGGTCG |
| CfCYP76AH16 | | CYP76AH16.qPCR.FOR1 | GGAGTTGTTTAGCGGGCCAGCG |
|  | | CYP76AH16.qPCR.REV1 | AGTGAAGACTCACGAATGCGATACATG |
| CfCYP76AH17 | | CYP76AH17.qPCR.FOR1 | TCTGCAAGCTGTCATCAAAGAATC |
|  | | CYP76AH17.qPCR.REV1 | AATCCAAACAACTCTCCTTTGGC |
| CfACT1-6 | | ACT1-6.qPCR.For1 | TGGAACGCGCCAAACATGGC |
|  | | ACT1-6.qPCR.Rev1 | TGCAGCTCCATCATCTCCTCGG |
| CfACT1-8 | | ACT1-8.qPCR.For1 | CTGGAACGCGCCAAAACAAGAG |
|  | | ACT1-8.qPCR.Rev1 | GCTCCTCGGCTTCTGCTGCGG |
| CfTIF4a | | CfTIF4a.For | CTATGATCTGCCAACTCAGCC |
|  | | CfTIF4a.Rev | CCTTGGTCACGAAGTTTATGG |
| CfEF1a | | CfEF1a.For | TGCATCACGAGGCTCTCCAG |
|  | | CfEF1a.Rev | GGCAACAAACCCACGCTTCA |
| **For USER cloning** | | | |
| **Gene Name** | **primer name** | | **sequence** |
| CfCYP707A90 | CfCYP707A90_5' | | GGCTTAAUATGATGGAACTCATCTGTTTGTTGTTGA |
|  | CfCYP707A90_3' | | GGTTTAAUTTATCATTTCTTGAGAGAGATTTTGATGGGCA |
| CfCYP96A70 | CfCYP86B1_5' | | GGCTTAAUATGGAGTCATTTTCATTGCTGTATCCAG |
|  | CfCYP86B1_3' | | GGTTTAAUTTAAGCCCACCTCTTGCTAACCCTAACCTTCAA |
| CfCYP89A110 | CfCYP89A110_5' | | GGCTTAAUATGATGATGAATTATCCACCCATTTTCG |
|  | CfCYP89A110_3' | | GGTTTAAUTTATTAACTTGTTCTGGGAGAGATGCGGGCAC |
| CfCYP76AH11 | CfCYP76AH11_5' | | GGCTTAAUATGATGGAGTTGGTGCAAGTGATTGCGG |
|  | CfCYP76AH11_3' | | GGTTTAAUTTATCAAATAGAAGGTGGGTTGAGTGGGATGAG |
| CYP76AH10 | CfCYP76AH1_5' | | GGCTTAAUATGGATAACTTTGTGCTCCTCGTTCTTC |
|  | CfCYP76AH1_3' | | GGTTTAAUTTAGAGGCTGATGGGGGTGGCTC |
| CYP76AH9 | CfCYP76AH2_5' | | GGCTTAAUATGGATTTCTTCACTCTTCTCGCTGCTC |
|  | CfCYP76AH2_3' | | GGTTTAAUTTAAGGTTGGATGGGATAGGCCCTGAGAGGAAC |
| CYP76AH8 | CfCYP76AH3_5' | | GGCTTAAUATGGAAACCATCACTCTTCTCCTTGCTC |
|  | CfCYP76AH3_3' | | GGTTTAAUTTATGGCTTAAGTGGAATGATCCTGAGAGGAGT |
| CfCYP71AT86 | CfCYP71AT86_5' | | GGCTTAAUATGATGATAATTTATTTCTTGATAATTC |
|  | CfCYP71AT86_3' | | GGTTTAAUTTACTAACCAACATATTTCTTAGCTAAAAGAAG |
| CfCYP71AT85 | CfCYP71AT85_5' | | GGCTTAAUATGATGATTTTTCTCTTCATGTCATTAT |
|  | CfCYP71AT85_3' | | GGTTTAAUTTACTAAAATATATATTTCGTAGGGACAAGCAC |
| CfCYP71D382 | CfCYP71BE8_5' | | GGCTTAAUATGATGGAGTCTCTCACTCTTGCTCTCT |
|  | CfCYP71BE8_3' | | GGTTTAAUTTATCACTCAACGACCTTGAGAGGCCTTGCGAG |
| CfCYP71AU43 | CfCYP71A1_5' | | GGCTTAAUATGATGCTTCTGTTGAATCCGTTTCTGC |
|  | CfCYP71A1_3' | | GGTTTAAUTTATCAGATGCGAGTTGCAACAGCGAGAAGAGG |
| CfCYP71AT87 | CfCYP83B4_5' | | GGCTTAAUATGATGATGCAGTTGGTCGCAGCACCAC |
|  | CfCYP83B4_3' | | GGTTTAAUTTATCACATAATGGCTGAGGATGGTGGAAGGTA |
| CfCYP728B26 | CfCYP716B1_5' | | GGCTTAAUATGATGATCATGATCTCCCTCCTCTGCT |
|  | CfCYP716B1_3' | | GGTTTAAUTTATTATAACAGTTTGTCTCTATGTTGCAACAG |
| CfCYP716A67 | CfCYP716A2_5' | | GGCTTAAUATGGAAATGGAAGTATTGTTATGCCAGG |
|  | CfCYP716A2_3' | | GGTTTAAUTTACAATTTGTGTAAAATCAAGTGAGCACCATG |
| CfCYP716A68 | CfCYP716A3_5' | | GGCTTAAUATGGGATCACACAAACAAATTATCAAAG |
|  | CfCYP716A3_3' | | GGTTTAAUTTAAGCTTGGTGGGGGTAGAGC |
| CfCYP716A69 | CfCYP716A4_5' | | GGCTTAAUATGGTGACGCGATGGGAAAGGTC |
|  | CfCYP716A4_3' | | GGTTTAAUTTAAGGGTGGTTGTGGGGGAAGAGT |
| CfCYP716C10 | CfCYP716C1_5' | | GGCTTAAUATGGAAATCTTGACGCTTGCCGTTTCCT |
|  | CfCYP716C1_3' | | GGTTTAAUTTAATGGTGGTAGAGGCGGACTGGC |
| CfCYP716D20 | CfCYP716D1_5' | | GGCTTAAUATGATGGAGGCCATTTGGATCATCACTT |
|  | CfCYP716D1_3' | | GGTTTAAUTTATTAACGAGAGTGAATACGAATGGGAAGTCC |
| CfCYP71D379 | CfCYP71D3 _5' | | GGCTTAAUATGATGGCCATTCAGTTTGCCTCCAACT |
|  | CfCYP71D3 _3' | | GGTTTAAUTTATCATCCTGAGACAGGAAGGTACGGTTCAGC |
| CfCYP71D383 | CfCYP71BE2_5' | | GGCTTAAUATGatGGAGTTTGAGTTGGCATCATCAT |
|  | CfCYP71BE2_3' | | GGTTTAAUTTATCAGGCGTGCAAAGGGCGCTTAAGAGTGGG |
| CfCYP71D381 | CfCYP71BE3_5' | | GGCTTAAUATGGAGTTCGATTTCCCGTCAGCACTCA |
|  | CfCYP71BE3_3' | | GGTTTAAUTTATGGAGCAAGTCGCAAAGGCCTCTTGAG |
| CfCYP71BE32 | CfCYP71BE7_5' | | GGCTTAAUATGGAGATCCAGTTTCCATCTCCACTGC |
|  | CfCYP71BE7_3' | | GGTTTAAUTTAGACAGGCAAAGGCCTCTTGATGATGGCAAC |
| CfCYP71D377 | CfCYP71D5_5' | | GGCTTAAUATGATGGAGCTGCCCTTGAGCTTCACCA |
|  | CfCYP71D5_3' | | GGTTTAAUTTATTAAGATGAGATAGGAGCGGTTGGGACCAC |
| CfCYP72A293 | CfCYP72A1_5' | | GGCTTAAUATGATGGAGATATTATACAGTTGGGTGG |
|  | CfCYP72A1_3' | | GGTTTAAUTTACTAATGGAGTGCGTGGAGGATCAAGGGGGC |
| CfCYP72A294 | CfCYP72A2_5' | | GGCTTAAUATGATGGAAATGGAAGTATTGTTATGCC |
|  | CfCYP72A2_3' | | GGTTTAAUTTATTACAATTTGTGTAAAATCAAGTGAGCACC |
| CfCYP82D63 | CfCYP82D1_5' | | GGCTTAAUATGATGGAATTCCACTACACAATCTATG |
|  | CfCYP82D1_3' | | GGTTTAAUTTACTAAACATCAATTTCCAAATTTAGCTAATC |
| CfCYP76AH16 | CfCYP76AH16_5' | | GGCTTAAUATGATGGAGTTGGTGGAAGTGATTGTGG |
|  | CfCYP76AH16_3' | | GGTTTAAUTTACAAAATGAAGGGTTGAGTGGGATGAGGTTG |
| CfCYP76AH15 | CfCYP76AH15_5' | | GGCTTAAUATGATGGAAACCATGACTCTTCTCCTCC |
|  | CfCYP76AH15_3' | | GGTTTAAUTTATCATGGCTTAAGTGGAATGATCCTGAGAGG |
| CfCYP76AH17 | CfCYP76AH17_5' | | GGCTTAAUATGATGGAAAGCATGAATGCTCTTGTCG |
|  | CfCYP76AH17_3' | | GGTTTAAUTTATTAAGGCTGAAGTGGAATGATCCTGAGGGG |
| CfCYP71D380 | CYP71D380_5' | | GGCTTAAUATGGATTTCTCATTTTTCTGGAACTTCA |
|  | CYP71D380_3' | | GGTTTAAUTTAAGGCGACTGCTTGTAAGGGGTGG |
| CfCYP716E1 | CfCYP716E1_5' | | GGCTTAAUATGATGCCTTGTCTTCTTCTTTTTCTTG |
|  | CfCYP716E1_3' | | GGTTTAAUTTACTATTTTGGATGTGGGCGGAGGTGAAGTGG |
| RoCYP76AH4 | RoCYP76AH4_5' | | GGCTTAAUATGATGGATTCCTTTCCTCTTCTCGTTG |
|  | RoCYP76AH4_3' | | GGTTTAAUTTATCAAGACTTAACTATTGGGATAATCCTAAG |
| CfACT1-6 | CfACT1-6_5' | | GGCTTAAUATGATGAAGGTGGAAAGATTTAGCAGGA |
|  | CfACT1-6_3' | | GGTTTAAUTTATTAGGTAGTAAATAATTTGATTTCTTCATC |
| CfACT1-7 | CfACT1-7_5' | | GGCTTAAUATGATGAAGGTGGAAAGATTTAGCAGGA |
|  | CfACT1-7_3' | | GGTTTAAUTTATTAGGTAGTAAATAATTTGATTTCTTCATC |
| CfACT1-8 | CfACT1-8_5' | | GGCTTAAUATGATGAAGGTTGAAAGAATAAGCAGGA |
|  | CfACT1-8_3' | | GGTTTAAUTTATTAGGTAGTAATTAATAATTTGATTTCTTC |
| CfACT2 | CfACT2 _5' | | GGCTTAAUATGATGATCCATTTACACAAAACAGTGT |
|  | CfACT2 _3' | | GGTTTAAUTTATTAGGTAGTAAATAATTTGATTTCTTCATC |
| CfACT3 | CfACT3_5' | | GGCTTAAUATGATGGAAGGCAAGGGTGTGGTTGTGG |
|  | CfACT3_3' | | GGTTTAAUTTATCAAGAGTTGGAAGTGGGGATGAGTTGGAA |
| CfACT4 | CfACT4_5' | | GGCTTAAUATGATGAAGGTGGAGATAGAGAGCCAAA |
|  | CfACT4_3' | | GGTTTAAUTTATTAGATGTATGTAGCTGCTGAAAGTTGAAG |
| CfACT5 | CfACT5_5' | | GGCTTAAUATGATGGAGATCGAAACCCAAGTTATCT |
|  | CfACT5_3' | | GGTTTAAUTTATTATGAAATCAAAGAAGTTTTGTGATGTGA |
| CfACT6 | CfACT6_5' | | GGCTTAAUATGATGGTCGGAAAAATGAAGGTGGAGG |
|  | CfACT6_3' | | GGTTTAAUTTATCAGCCGGTGAGGATGCGAAGCTCCTCATC |
| CfACT7 | CfACT7_5' | | GGCTTAAUATGATGGCGACAGTCGAAACGATATCCA |
|  | CfACT7_3' | | GGTTTAAUTTATTAATTATGAAGTTCAAAAATGTCATCACT |
| CfACT8 | CfACT8_5' | | GGCTTAAUATGATGGAAACATTAAACGTCAAGGTTT |
|  | CfACT8_3' | | GGTTTAAUTTATCACAGCGCCGATGTGATGAACACTCCGGT |
| **For 5'RACE cloning** | | | |
| **Gene Name** | **primer name** | | **sequence** |
| CfCYP76AH15 | 5RACE_AH5.1 | | GCCTGTGCGATGGTTCTCCCGGAAAAC |
|  | 5RACE_AH5.2 | | GCTATCACGGTGTATACACTCCCGAGTTGGAC |
| CfCYP76AH17 | 5RACE_AH7.1 | | GTCTCCGAAATTAGCCACGCCCACAATCG |
|  | 5RACE_AH7.2 | | GGCATCTCCGACTCATCCACCAGTTTCCTC |
| CfACT1-8 | 5R.4-3A.2 | | GCCTTTGTTCTCTCACGCATGTTGATCCC |
|  | 5R.4-3A.3 | | ACTCTGCAGCTCCTCGGCTTCTGCTGCGG |
|  |  | |  |
| **Cloning of pseudomature diTPS, CYP and ACT genes for introduction into *S. cerevisae*** | | | |
| **Gene Name** | **primer name** | | **sequence** |
| CfTPS2 | CfTPS2_COfwdU | | AGCGATACGUAAAAATGTCCAGAGTTGCTTCCTTGG |
|  | CfTPS2_COrevU | | CACGCGAUTTAAACAACAGGTTCGAACAAAACTTGG |
| CfTPS3 | CfTPS3_COfwdU | | ATCAACGGGUAAAAATGATCACCTCCAAATCTTCC |
|  | CfTPS3_COrevU | | CGTGCGAUTCAGTTACTGACGCAGGAC |
| CfTPS1 | CfTPS1_COfwdU | | AGCGATACGUAAAATGGCTTGGATGAACAACGGTAAG |
|  | CfTPS1_COrevU | | CACGCGAUTCAGGCGACTGGTTCGAAC |
| CfCPR | CfCPR_COfwdU | | AGCGATACGUAAAAATGGAATCCACCATCGAAAAGTTGTCC |
|  | CfCPR_COrevU | | CACGCGAUTTACCAAACATCTCTTAAGTATCTACC |
| CfCYP76AH8 | CfCYP76AH8_COfwdU | | AGCGATACGUAAAAATGGAAACCATCACCTTGTTGTTGG |
|  | CfCYP76AH8_COrevU | | CACGCGAUTTATGGTTTCAATGGAATGATTCTC |
| CfCYP76AH11 | CfCYP76AH11_COfwdU | | ATCAACGGGUAAAAATGGAATTGGTCCAAGTTATCGC |
|  | CfCYP76AH11_COrevU | | CGTGCGAUTCAGATGGATGGTGGGTTCAAAGG |
| CfCYP76AH8 | CfCYP76AH8_COfwdU | | ATCAACGGGUAAAAATGGAAACCATCACCTTGTTGTTGG |
|  | CfCYP76AH8_COrevU | | CGTGCGAUTTATGGTTTCAATGGAATGATTCTC |
| CfCYP76AH16 | CfCYP76AH16_COfwdU | | CGTGCGAUTTAAAAAGATGGGTTCAATGGGATC |
|  | CfCYP76AH16_COrevU | | ATCAACGGGUAAAAATGGAATTGGTCGAAGTTATCG |
| CYP76AH11 | CYP76AH11fwdUtef | | AGCGATACGUAAAAATGGAATTGGTCCAAGTTATCGC |
|  | CYP76AH11revUtef | | CACGCGAUTCAGATGGATGGTGGGTTCAAAGG |
| CYP76AH16 | CYP76AH16fwdUtef | | AGCGATACGUAAAAATGGAATTGGTCGAAGTTATCG |
|  | CYP76AH16revUtef | | CACGCGATTTAAAAAGATGGGTTCAATGGGATC |
| CfCYP76AH9 | CYP76AH9fwd | | CGTGCGAUTTATGGTTGAATTGGATAAGC |
|  | CYP76AH9rev | | ATCAACGGGUAAAAATGGACTTCTTCACTTTGTTGG |
| CfCYP76AH10 | CYP76AH10fwd | | CGTGCGAUTCACAAAGAAATTGGGGTAGC |
|  | CYP76AH10rev | | ATCAACGGGUAAAAATGGACAACTTCGTTTTGTTGG |
| CfCYP76AH15-CO | CYP76AH15fwd | | CGTGCGAUTCAAGGCTTCAATGGGATAATTCTC |
|  | CYP76AH15rev | | ATCAACGGGUAAAAATGGAAACCATGACCTTGTTGTTGC |
| CfCYP76AH17-CO | CYP76AH17fwd | | CGTGCGAUTTATGGTTGCAATGGAATAATTCTC |
|  | CYP76AH17rev | | ATCAACGGGUAAAAATGGAATCCATGAACGCCTTGGTTG |
| CfACT1 | CfACT1fwdU | | AGCGATACGUAAAAATGAAGGTCGAAAGATTCTCCAG |
|  | CfACT1revU | | CACGCGAUTCAGGTGGTGAACAACTTGATTTC |
| CfACT2 | CfACT2fwdU | | AGCGATACGUAAAAATGATCCACTTGCACAAGACC |
|  | CfACT2revU | | CACGCGAUTCAGGTGGTGAACAACTTGATTTC |
| CfACT3 | CfACT3fwdU | | AGCGATACGUAAAAATGGAAGGTAAGGGTGTTGTCG |
|  | CfACT3revU | | CACGCGAUTTAAGAGTTGGAGGTTGGAATC |
| CfACT4 | CfACT4fwdU | | AGCGATACGUAAAAATGAAGGTCGAAATCGAATCC |
|  | CfACT4revU | | CACGCGAUTCAAATGTAGGTAGCAGCAGAC |
| CfACT5 | CfACT5fwdU | | AGCGATACGUAAAAATGGAAATCGAAACCCAAGTC |
|  | CfACT5revU | | CACGCGAUTTAAGAGATCAATGAGGTCTTATG |
| CfACT6 | CfACT6fwdU | | AGCGATACGUAAAAATGGTCGGTAAGATGAAGGTCG |
|  | CfACT6revU | | CACGCGAUTTAACCAGTCAAGATTCTCAATTC |
| CfACT7 | CfACT7fwdU | | AGCGATACGUAAAAATGGCTACCGTTGAAACC |
|  | CfACT7revU | | CACGCGAUTTAGTTATGCAATTCAAAGATGTC |
| CfACT8 | CfACT8fwdU | | AGCGATACGUAAAAATGGAAACCTTGAACGTC |
|  | CfACT8revU | | CACGCGAUTCACAAAGCAGAAGTAATG |
| CfACT1-6 | CfACT1-6fwdU | | AGCGATACGUAAAAATGAAGGTCGAAAGATTCTCC |
|  | CfACT1-6revU | | CACGCGAUTCAGGTGGTGAACAACTTGATTTCTTCG |
| CfACT1-8 | CfACT1-8fwdU | | AGCGATACGUAAAAATGAAGGTCGAAAGAATCTCCAG |
|  | CfACT1-8revU | | CACGCGAUTCAGGTGGTGATCAACAACTTGATTTCTTCG |
| CfACT1-3 | CfACT1-3fwd | | AGCGATACGUAAAAATGAAGGTCGAAAGATTCTCC |
|  | CfACT1-3rev | | CACGCGAUTCAGGTGGTGATCAACAACTTG |

**References**

Andersen-Ranberg, J, KT Kongstad, MT Nielsen, NB Jensen, I Pateraki, SS Bach, B Hamberger, P Zerbe, D Staerk, J Bohlmann, BL Møller B Hamberger. 2016. Expanding the landscape of diterpene structural diversity through stereochemically controlled combinatorial biosynthesis. *Angewandte Chemie International Edition* **55:** 2142-2146.

Asada, Y, W Li, T Terada, X Kuang, Q Li, T Yoshikawa, S Hamaguchi, I Namekata, H Tanaka K Koike. 2012. Labdane-type diterpenoids from hairy root cultures of *Coleus forskohlii*, possible intermediates in the biosynthesis of forskolin. *Phytochemistry* **79:** 141-146.

Gabetta, B, G Zini B Danieli. 1989. Minor diterpenoids of *Coleus forskohlii*. *Phytochemistry* **28:** 859-862.

Nour-Eldin, HH, BG Hansen, MHH Nørholm, JK Jensen BA Halkier. 2006. Advancing uracil-excision based cloning towards an ideal technique for cloning PCR fragments. *Nucleic Acids Research* **34:** e122.
