# Supplementary material for: Total biosynthesis of the cyclic AMP booster forskolin from Coleus forskohlii
Source: eLife. 2017 Mar 14;6:e23001. doi: 10.7554/eLife.23001 (PMC5388535; doi:10.7554/eLife.23001)
Supplement: Figure 4—source data 1. — DOI: http://dx.doi.org/10.7554/eLife.23001.012 [file elife-23001-fig4-data1.docx]

**Figure 4-source data 1.**

NMR spectra’s of selected 13R-manoyl oxide derived molecules**.**

|  |
| --- |
| Figure S1A. ^1^H spectrum of 19-hydroxy-13R-manoyl oxide (**3c**) |

|  |
| --- |
| Figure S1B. ^13^C spectrum of 19-hydroxy-13R-manoyl oxide (**3c**) |

|  |
| --- |
| Figure S1B. HSQC spectrum of 19-hydroxy-13R-manoyl oxide (**3c**) |

|  |
| --- |
| Figure S1C. HMBC spectrum of 19-hydroxy-13R-manoyl oxide (**3c**) |

|  |
| --- |
| Figure S1D. NOESY spectrum of 19-hydroxy-13R-manoyl oxide (**3c**) |

|  |
| --- |
| Figure S1E. COSY spectrum of 19-hydroxy-13R-manoyl oxide (**3c**) |

|  |
| --- |
| Figure S2A. ^1^H spectrum of 2-hydroxy-13R-manoyl oxide (**3b**) |

|  |
| --- |
| Figure S1B. ^13^C spectrum of 19-hydroxy-13R-manoyl oxide (**3c**) |

|  |
| --- |
| Figure S2B. HSQC spectrum of 2-hydroxy-13R-manoyl oxide (**3b**) |

|  |
| --- |
| Figure S2C. HMBC spectrum of 19-hydroxy-13R-manoyl oxide (**3b**) |

|  |
| --- |
| Figure S1D. NOESY spectrum of 2-hydroxy-13R-manoyl oxide (**3b**) |

|  |
| --- |
| Figure S2E. COSY spectrum of 2-hydroxy-13R-manoyl oxide (**3b**) |

|  |
| --- |
| Figure S3A. ^1^H spectrum of 1,11-dihydroxy-13R-manoyl oxide (**5d**) |

|  |
| --- |
| Figure S1B. ^13^C spectrum of 19-hydroxy-13R-manoyl oxide (**3c**) |

|  |
| --- |
| Figure S3B. HSQC spectrum of 1,11-dihydroxy-13R-manoyl oxide (**5d**) |

|  |
| --- |
| Figure S3C. HMBC spectrum of 1,11-dihydroxy-13R-manoyl oxide (**5d**) |

|  |
| --- |
| Figure S3D. NOESY spectrum of 1,11-dihydroxy-13R-manoyl oxide (**5d**) |

|  |
| --- |
| Figure S3E. COSY spectrum of 1,11-dihydroxy-13R-manoyl oxide (**5d**) |

|  |
| --- |
| Figure S4A. ^1^H spectrum of 9-deoxydeacetylforskolin (**10b**) |

|  |
| --- |
| Figure S4B. ^13^C spectrum of 9-deoxydeacetylforskolin (**10b**) |

|  |
| --- |
| Figure S4C. HSQC spectrum of 9-deoxydeacetylforskolin (**10b**) |

|  |
| --- |
| Figure S4D. HMBC spectrum of 9-deoxydeacetylforskolin (**10b**) |

|  |
| --- |
| Figure S4E. NOESY spectrum of 9-deoxydeacetylforskolin (**10b**) |

|  |
| --- |
| Figure S4F. COSY spectrum of 9-deoxydeacetylforskolin (**10b**) |

|  |
| --- |
| Figure S5A. ^1^H spectrum of 1,9-dideoxydeacetylforskolin (**7h**) |

|  |
| --- |
| Figure S5B. ^13^C spectrum of 1,9-dideoxydeacetylforskolin (**7h**) |

|  |
| --- |
| Figure S5C. HSQC spectrum of 1,9-dideoxydeacetylforskolin (**7h**) |

|  |
| --- |
| Figure S5D. HMBC spectrum of 1,9-dideoxydeacetylforskolin (**7h**) |

|  |
| --- |
| Figure S5E. NOESY spectrum of 1,9-dideoxydeacetylforskolin (**7h**) |

|  |
| --- |
| Figure S5F. COSY spectrum of 1,9-dideoxydeacetylforskolin (**7h**) |

|  |
| --- |
| Figure S6A. ^1^H spectrum of coloerol (**3a**) |

|  |
| --- |
| Figure S6B. ^13^C spectrum of coloerol (**3a**) |

|  |
| --- |
| Figure S6C. HSQC spectrum of coloerol (**3a**) |

|  |
| --- |
| Figure S6D. HMBC spectrum of coloerol (**3a**) |

|  |
| --- |
| Figure S6E. ROESY spectrum of coloerol (**3a**) |

|  |
| --- |
| Figure S6F. COSY spectrum of coloerol (**3a**) |
