# Supplementary material for: Total biosynthesis of the cyclic AMP booster forskolin from Coleus forskohlii
Source: eLife. 2017 Mar 14;6:e23001. doi: 10.7554/eLife.23001 (PMC5388535; doi:10.7554/eLife.23001)
Supplement: Supplementary file 1. — GC-MS and LC-qTOF-MS chromatograms of the identified diterpenoids are shown in previous figures. DOI: http://dx.doi.org/10.7554/eLife.23001.025 [file elife-23001-supp1.docx]

**Supplementary file 1.**

Overview of 13*R*-manoyl oxide-derived diterpenoids identified in *N. benthamiana,* expressing combinations of *C. forskohlii* genes encoding CYPs and acetyltransferases together with genes encoding the required enzymes for biosynthesis of 13*R*-manoyl oxide (*Cf*DXS, *Cf*GGPPs, *Cf*TP2 and *Cf*TPS3). GC-MS and LC-qTOF-MS chromatograms of the identified diterpenoids are shown in previous figures.

|  |  |  |  |  |  |  |  | **Single CYPs** | | | | | | **Two CYPs** | | | | | | **Three CYPs** | | | **ACTs** | | **Standards** | | | | | |
| --- | --- | --- | --- | --- | --- | --- | --- | --- | --- | --- | --- | --- | --- | --- | --- | --- | --- | --- | --- | --- | --- | --- | --- | --- | --- | --- | --- | --- | --- | --- |
|  | # |  |  |  |  |  | (-) | CYP76AH15 | CYP76AH8 | CYP76AH17 | CYP76AH11 | CYP76AH16 | CYP71D781 | CYP76AH15+CYP76AH11 | CYP76AH8+CYP76AH11 | CYP76AH17+CYP76AH11 | CYP76AH15+CYP76AH16 | CYP76AH8+CYP76AH16 | CYP76AH17+CYP76AH16 | CYP76AH15+CYP76AH11+CYP76AH16 | CYP76AH8+CYP76AH11+CYP76AH16 | CYP76AH17+CYP76AH11+CYP76AH16 | CYP76AH15+CYP76AH11+CYP76AH16+ACT1-6 | CYP76AH15+CYP76AH11+CYP76AH16+ACT1-8 | 11-oxo-13*R*-manoyl oxide STD (2) | 9-hydroxy-13*R*-manoyl oxide STD (3a) | 1,11-dihydroxy-13*R*-manoyl oxide(5d)  1,9-dideoxydeacetylforskolin (7h) STD mix | 9-deoxydeacetylforskolin STD (10b) | Deacetylforskolin STD (13b) | Forskolin STD (16c) |
| **GC-MS** | **1** | 13*R*-(+)-manoyl oxide | | |  |  | x |  |  |  |  |  |  |  |  |  |  |  |  |  |  |  |  |  |  |  |  |  |  |  |
|  | **2** | 11-oxo-13*R*-manoyl oxide | | |  |  |  | x | x | x |  |  |  |  |  |  |  |  |  |  |  |  |  |  | x |  |  |  |  |  |
|  | **3a** | 9-hydroxy-13*R*-manoyl oxide | | |  |  |  |  |  |  |  | x |  |  |  |  |  |  |  |  |  |  |  |  |  | x |  |  |  |  |
|  | **3b** | 2-hydroxy-13*R*-manoyl oxide | | |  |  |  |  |  |  |  |  | x |  |  |  |  |  |  |  |  |  |  |  |  |  |  |  |  |  |
|  | **3c** | 19-hydroxy-13*R*-manoyl oxide | | | |  |  |  |  |  |  |  | x |  |  |  |  |  |  |  |  |  |  |  |  |  |  |  |  |  |
|  |  | **MO deriv.** | **MF** | **RT [min]** | **Mw [Da]** | ***m*/*z*[M+Na]** | | **ΔM (amu.)** | | | | | | | | | | | | | | | | | | | | | | |
| **LC- qTOF-MS** | **4a** | MO(O)(OH) | C_20_H_32_O_3_ | 19.66 | ###### | ###### |  |  |  |  | # |  |  |  |  |  |  |  |  |  |  |  |  |  |  |  |  |  |  |  |
|  | **4b** | MO(O)(OH) | C_20_H_32_O_3_ | 22.50 | ###### | ###### |  |  |  |  | # |  |  |  |  |  |  |  |  |  |  |  |  |  |  |  |  |  |  |  |
|  | **4c** | MO(O)(OH) | C_20_H_32_O_3_ | 24.90 | ###### | ###### |  | tr | # | 3.5 |  |  |  |  | # | # |  | 1.5 | 1.5 |  |  | # |  |  |  |  |  |  |  |  |
|  | **4d** | MO(O)(OH) | C_20_H_32_O_3_ | 26.63 | ###### | ###### |  |  |  |  |  | 1.5 |  |  |  |  |  |  |  |  |  |  |  |  |  |  |  |  |  |  |
|  | **5a** | MO(OH)_2_ | C_20_H_34_O_3_ | 16.46 | ###### | ###### |  |  |  |  | # |  |  |  |  | # |  | # | # |  |  |  |  |  |  |  |  |  |  |  |
|  | **5b** | MO(OH)_2_ | C_20_H_34_O_3_ | 18.90 | ###### | ###### |  |  |  |  | # |  |  |  |  |  |  |  |  |  |  |  |  |  |  |  |  |  |  |  |
|  | **5c** | MO(OH)_2_ | C_20_H_34_O_3_ | 21.20 | ###### | ###### |  |  |  |  | 3.1 |  |  |  |  |  |  |  |  |  |  |  |  |  |  |  |  |  |  |  |
|  | **5d** | MO(OH)_2_ | C_20_H_34_O_3_ | 22.28 | ###### | ###### |  | tr | # | # |  |  |  | # | 1.1 | # |  |  | # |  | # | # |  |  |  |  | 1.2 |  |  |  |
|  | **5e** | MO(OH)_2_ | C_20_H_34_O_3_ | 27.50 | ###### | ###### |  |  |  |  |  | 3.7 |  |  |  |  | # | # | # |  |  |  |  |  |  |  |  |  |  |  |
|  | **6a** | MO(O)_2_(OH) | C_20_H_30_O_4_ | 14.30 | ###### | ###### |  |  |  |  | 1.5 |  |  | 1.1 | -1.1 | -1.1 |  |  |  | # | # | # |  |  |  |  |  |  |  |  |
|  | **6b** | MO(O)_2_(OH) | C_20_H_30_O_4_ | 21.10 | ###### | ###### |  |  |  |  |  |  |  |  |  |  | 3.1 | # | 1.4 | tr | # | # |  |  |  |  |  |  |  |  |
|  | **7a** | MO(O)(OH)_2_ | C_20_H_32_O_4_ | 12.50 | ###### | ###### |  |  | # | 1.3 |  |  |  | tr | tr | # |  |  | # | # | # | 1.5 |  |  |  |  |  |  |  |  |
|  | **7b** | MO(O)(OH)_2_ | C_20_H_32_O_4_ | 12.75 | ###### | ###### |  |  | 1.6 | # |  |  |  | # | # | # |  | # | # | # | # | # |  |  |  |  |  |  |  |  |
|  | **7d** | MO(O)(OH)_2_ | C_20_H_32_O_4_ | 14.30 | ###### | ###### |  |  |  |  | 1.8 |  |  |  |  |  |  |  |  |  |  |  |  |  |  |  |  |  |  |  |
|  | **7e** | MO(O)(OH)_2_ | C_20_H_32_O_4_ | 15.10 | ###### | ###### |  |  |  |  | # |  |  |  |  |  |  |  |  |  |  |  |  |  |  |  |  |  |  |  |
|  | **7f** | MO(O)(OH)_2_ | C_20_H_32_O_4_ | 15.60 | ###### | ###### |  |  | # | 1.7 |  |  |  | # | # | # | # | # | # | tr |  | 1.4 |  |  |  |  |  |  |  |  |
|  | **7g** | MO(O)(OH)_2_ | C_20_H_32_O_4_ | 18.20 | ###### | ###### |  |  |  |  |  |  |  |  |  |  |  | # | 1.2 |  | # | # |  |  |  |  |  |  |  |  |
|  | **7h** | MO(O)(OH)_2_ | C_20_H_32_O_4_ | 21.30 | ###### | ###### |  |  |  |  |  |  |  | -1.1 | 1.2 | # |  |  |  | # | 3.7 | # |  |  |  |  | 1.3 |  |  |  |
|  | **7i** | MO(O)(OH)_2_ | C_20_H_32_O_4_ | 23.67 | ###### | ###### |  |  |  |  |  |  |  |  |  |  |  | # | # |  | # | # |  |  |  |  |  |  |  |  |
|  | **8a** | MO(OH)_3_ | C_20_H_34_O_4_ | 10.69 | ###### | ###### |  |  |  |  | 1.6 |  |  |  |  |  |  |  |  |  |  |  |  |  |  |  |  |  |  |  |
|  | **8b** | MO(OH)_3_ | C_20_H_34_O_4_ | 11.10 | ###### | ###### |  |  | # | 1.1 |  |  |  |  |  |  |  |  |  |  |  |  |  |  |  |  |  |  |  |  |
|  | **8c** | MO(OH)_3_ | C_20_H_34_O_4_ | 11.30 | ###### | ###### |  |  |  |  | # |  |  |  | # | 0.7 |  |  | 2.1 |  | # | 3.5 |  |  |  |  |  |  |  |  |
|  | **8d** | MO(OH)_3_ | C_20_H_34_O_4_ | 18.35 | ###### | ###### |  |  |  |  |  |  |  |  |  |  |  |  |  |  | # | # |  |  |  |  |  |  |  |  |
|  | **8e** | MO(OH)_3_ | C_20_H_34_O_4_ | 22.58 | ###### | ###### |  |  |  |  |  |  |  |  |  |  |  |  |  |  |  | # |  |  |  |  |  |  |  |  |
|  | **8f** | MO(OH)_3_ | C_20_H_34_O_4_ | 24.00 | ###### | ###### |  |  |  |  |  |  |  |  |  |  |  |  | # |  |  |  |  |  |  |  |  |  |  |  |
|  | **8g** | MO(OH)_3_ | C_20_H_34_O_4_ | 26.60 | ###### | ###### |  |  |  |  |  |  |  | # | # | # |  |  |  |  |  |  |  |  |  |  |  |  |  |  |
|  | **9a** | MO(O)_2_(OH)_2_ | C_20_H_30_O_5_ | 9.70 | ###### | 373.19855 |  |  |  |  | # |  |  | # | 0.5 | # |  |  |  | tr |  | 1.5 |  |  |  |  |  |  |  |  |
|  | **9b** | MO(O)_2_(OH)_2_ | C_20_H_30_O_5_ | 13.79 | ###### | 373.19855 |  |  |  |  |  |  |  |  |  |  | # | 1.0 | # | # | # | # |  |  |  |  |  |  |  |  |
|  | **10a** | MO(O)(OH)_3_ | C_20_H_32_O_5_ | 5.50 | ###### | ###### |  |  |  |  |  |  |  |  | # | # |  |  |  |  |  |  |  |  |  |  |  |  |  |  |
|  | **10b** | MO(O)(OH)_3_ | C_20_H_32_O_5_ | 9.00 | ###### | ###### |  |  |  |  | 0.5 |  |  | -1.1 | # | # |  |  |  | # | # | # |  |  |  |  |  | 1.6 |  |  |
|  | **10c** | MO(O)(OH)_3_ | C_20_H_32_O_5_ | 14.25 | ###### | ###### |  |  |  |  |  |  |  |  |  |  |  |  |  | 0.5 | # | 4.7 |  |  |  |  |  |  |  |  |
|  | **10d** | MO(O)(OH)_3_ | C_20_H_32_O_5_ | 14.77 | ###### | ###### |  |  |  |  |  |  |  |  |  |  | # | 4.5 | # | 0.5 | # | # |  |  |  |  |  |  |  |  |
|  | **10e** | MO(O)(OH)_3_ | C_20_H_32_O_5_ | 20.80 | ###### | ###### |  |  |  |  |  |  |  |  |  |  |  |  |  | # | # | # |  |  |  |  |  |  |  |  |
|  | **11a** | MO(OH)_4_ | C_20_H_34_O_5_ | 9.70 | ###### | ###### |  |  |  |  |  |  |  |  |  |  |  | # | 1.7 |  | # | # |  |  |  |  |  |  |  |  |
|  | **12a** | MO(O)_2_(OH)_3_ | C_20_H_30_O_6_ | 9.30 | ###### | ###### |  |  |  |  |  |  |  |  |  |  |  |  |  | # | # | # | # |  |  |  |  |  |  |  |
|  | **12b** | MO(O)_2_(OH)_3_ | C_20_H_30_O_6_ | 13.63 | ###### | ###### |  |  |  |  |  |  |  |  |  |  |  |  |  | 1.6 | # | 5.0 |  |  |  |  |  |  |  |  |
|  | **13a** | MO(O)(OH)_4_ | C_20_H_32_O_6_ | 9.65 | ###### | 391.20911 |  |  |  |  |  |  |  |  |  |  |  | # | 1.8 |  |  | # |  |  |  |  |  |  |  |  |
|  | **13b** | MO(O)(OH)_4_ | C_20_H_32_O_6_ | 11.16 | ###### | 391.20911 |  |  |  |  |  |  |  |  |  |  |  | tr | tr | # | # | # | # | # |  |  |  |  | 1.5 |  |
|  | **14a** | MO(O)(OH)_2_(Ac) | C_22_H_34_O_5_ | 19.87 | ###### | ###### |  |  |  |  |  |  |  |  |  |  |  |  |  |  |  |  | # |  |  |  |  |  |  |  |
|  | **15a** | MO(O)(OH)_3_(Ac) | C_22_H_34_O_6_ | 10.39 | ###### | 417.22475 |  |  |  |  |  |  |  |  |  |  |  |  |  |  |  |  | # |  |  |  |  |  |  |  |
|  | **15b** | MO(O)(OH)_3_(Ac) | C_22_H_34_O_6_ | 15.01 | ###### | 417.22475 |  |  |  |  |  |  |  |  |  |  |  |  |  |  |  |  | # | # |  |  |  |  |  |  |
|  | **16a** | MO(O)(OH)_4_(Ac) | C_22_H_34_O_7_ | 14.07 | ###### | ###### |  |  |  |  |  |  |  |  |  |  |  |  |  |  |  |  | # |  |  |  |  |  |  |  |
|  | **16b** | MO(O)(OH)_4_(Ac) | C_22_H_34_O_7_ | 15.47 | ###### | ###### |  |  |  |  |  |  |  |  |  |  |  |  |  |  |  |  | # |  |  |  |  |  |  |  |
|  | **16c** | MO(O)(OH)_4_(Ac) | C_22_H_34_O_7_ | 18.30 | ###### | ###### |  |  |  |  |  |  |  |  |  |  |  |  |  |  |  |  | # | # |  |  |  |  |  | 0.5 |
|  | **17a** | MO(O)(OH)_2_(Ac)_2_ | C_24_H_36_O_6_ | 24.04 | ###### | ###### |  |  |  |  |  |  |  |  |  |  |  |  |  |  |  |  | # |  |  |  |  |  |  |  |
|  | **18a** | MO(O)(OH)_4_(Ac)_2_ | C_24_H_36_O_8_ | 21.26 | ###### | ###### |  |  |  |  |  |  |  |  |  |  |  |  |  |  |  |  |  | # |  |  |  |  |  |  |
|  | **19a** | MO(O)(OH)_3_(Ac)_3_ | C_24_H_38_O_8_ | 21.66 | ###### | ###### |  |  |  |  |  |  |  |  |  |  |  |  |  |  |  |  | 0.7 |  |  |  |  |  |  |  |
